# Corrected and Republished from: “Understanding Lactobacillus paracasei and Streptococcus oralis Biofilm Interactions through Agent-Based Modeling”

**DOI:** 10.1128/msphere.00656-22

**Published:** 2023-03-21

**Authors:** Linda Archambault, Sherli Koshy-Chenthittayil, Angela Thompson, Anna Dongari-Bagtzoglou, Reinhard Laubenbacher, Pedro Mendes

**Affiliations:** a Center for Quantitative Medicine, University of Connecticut School of Medicine, Farmington, Connecticut, USA; b Department of Oral Health and Diagnostic Sciences, University of Connecticut School of Dental Medicine, Farmington, Connecticut, USA; c Department of Medicine, University of Florida, Gainesville, Florida, USA; d Center for Cell Analysis and Modeling, University of Connecticut School of Medicine, Farmington, Connecticut, USA; e Department of Cell Biology, University of Connecticut School of Medicine, Farmington, Connecticut, USA; The University of Iowa

**Keywords:** biofilm, computational modeling, agent-based modeling, *Streptococcus oralis*, *Lactobacillus paracasei*, oral health

## Abstract

As common commensals residing on mucosal tissues, *Lactobacillus* species are known to promote health, while some Streptococcus species act to enhance the pathogenicity of other organisms in those environments. In this study we used a combination of *in vitro* imaging of live biofilms and computational modeling to explore biofilm interactions between Streptococcus oralis, an accessory pathogen in oral candidiasis, and Lactobacillus paracasei, an organism with known probiotic properties. A computational agent-based model was created where the two species interact only by competing for space, oxygen, and glucose. Quantification of bacterial growth in live biofilms indicated that S. oralis biomass and cell numbers were much lower than predicted by the model. Two subsequent models were then created to examine more complex interactions between these species, one where *L. paracasei* secretes a surfactant and another where *L. paracasei* secretes an inhibitor of S. oralis growth. We observed that the growth of S. oralis could be affected by both mechanisms. Further biofilm experiments support the hypothesis that *L. paracasei* may secrete an inhibitor of S. oralis growth, although they do not exclude that a surfactant could also be involved. This contribution shows how agent-based modeling and experiments can be used in synergy to address multiple-species biofilm interactions, with important roles in mucosal health and disease.

**IMPORTANCE** We previously discovered a role of the oral commensal Streptococcus oralis as an accessory pathogen. S. oralis increases the virulence of Candida albicans infections in murine oral candidiasis and epithelial cell models through mechanisms which promote the formation of tissue-damaging biofilms. *Lactobacillus* species have known inhibitory effects on biofilm formation of many microbes, including Streptococcus species. Agent-based modeling has great advantages as a means of exploring multifaceted relationships between organisms in complex environments such as biofilms. Here, we used an iterative collaborative process between experimentation and modeling to reveal aspects of the mostly unexplored relationship between S. oralis and *L. paracasei* in biofilm growth. The inhibitory nature of *L. paracasei* on S. oralis in biofilms may be exploited as a means of preventing or alleviating mucosal fungal infections.

## INTRODUCTION

*Lactobacillus* and Streptococcus species are ubiquitous commensals found in the human oral cavity but also the genitourinary and gastrointestinal tracts. Mitis group streptococci (MGS), primarily represented by Streptococcus oralis, Streptococcus sanguinis, Streptococcus gordonii, and Streptococcus mitis, are prominent among the first colonizers of biofilms on mucosal and tooth surfaces (1, 2). MGS were originally found to play a positive role, maintaining microbiome homeostasis in the oral cavity by antagonizing other microbes such as the cariogenic Streptococcus mutans (3, 4). Although they are members of the healthy oral microbiota, MGS were more recently recognized for their role as accessory pathogens, enhancing the virulence of potentially harmful members of the microbiota, such as Candida albicans (5–8) and Porphyromonas gingivalis (9, 10). In addition, MGS can be pathogens in their own right: when they enter the bloodstream, they can cause endocarditis, bacteremia, and toxic shock (11–13).

Many *Lactobacillus* species have probiotic properties that promote gut, vaginal, and oral health (14–17). *Lactobacilli* possess a diverse array of mechanisms implicated in the inhibition of vital processes in other bacteria; these include inhibition of growth through production of lactic acid and bacteriocins and prevention of attachment to surfaces by competition, coaggregation, and production of biosurfactants, which may also promote biofilm dispersion (18–22). In the oral environment, *Lactobacillus* species inhibit the growth and biofilm formation of S. mutans via multiple mechanisms. For example, secreted molecules found in supernatants of *Lactobacillus* cultures inhibit growth, adhesion, and biofilm formation (23, 24), and cell wall component lipoteichoic acid interferes with S. mutans sucrose metabolism, reducing the production of exopolysaccharide, an important component of biofilms (25). *Lactobacillus* spp. also inhibit Streptococcus pyogenes hemolytic activity and adhesion to epithelial cells (26). In addition to the great variety of antimicrobial effects attributed to different *Lactobacillus* spp., a considerable genetic and phenotypic diversity exists in oral streptococcal species, and even strains within the same species, which affects growth in different oral ecological niches and their role as pathobionts (6). Lactobacillus paracasei is known to produce molecules with antimicrobial and surfactant properties (27–29), but interactions between *L. paracasei* and S. oralis in the biofilm growth form have never been explored.

To fully understand the complex community interactions between species, mathematical modeling is a complement to an experimental approach (30, 31). It helps consolidate data, and after validation, it can help in making predictions (32) and provide an integrative and quantitative understanding of the system studied. Agent-based models (ABM) are particularly suited to represent biofilms, as they capture the activities of each individual cell (autonomous agent) in the community (33). These models incorporate rules of growth, division, movement, and decay for each cell, and these rules can be deterministic or stochastic. The cells are embedded in a spatial environment with relevant physical constraints, such as diffusion of chemicals (34, 35). The behavior of each individual agent and the environmental constraints contribute to the emergence of the total population behavior, i.e., the biofilm development and structure. Very few agent-based models have been constructed using input both from the literature and experiments (36, 37). Our model is another addition to this small group of ABMs built through cross talk between experimentation and simulation.

This work aims to further our understanding of the interactions between S. oralis and *L. paracasei* during biofilm growth with a rarely used combination of agent-based modeling and experimentation. The agent-based model was a device to better understand the dual-species biofilm growth characteristics. The growth parameters of the model were estimated using the experimentally determined behavior of single-species biofilms and data from the literature. Live fluorescence imaging showed that the growth of S. oralis is inhibited in the dual biofilm with *L. paracasei*. We then constructed two models expressing two distinct hypotheses: noncompetitive inhibition and surfactant production. The models were validated with further experiments to explore the nature of the interactions between S. oralis and *L. paracasei*.

## RESULTS

### Model calibration.

**FIG 1:**
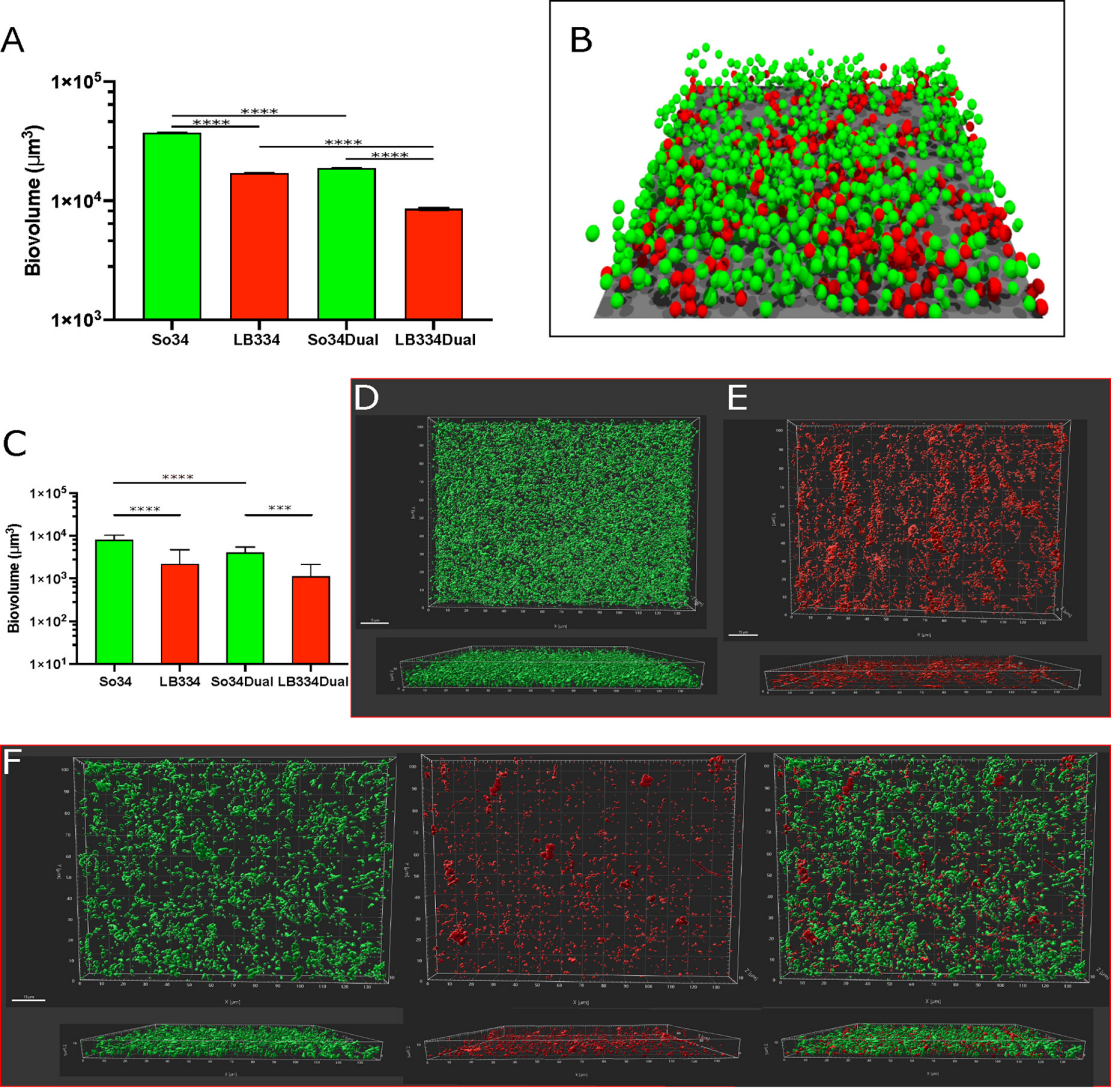
Comparison of simulated and *in vitro* biofilm growth. (A and B) Simulation of biofilm growth in a purely competitive model. (A) Biovolume for single and dual biofilms from simulations of 16-h growth. Results are expressed as the mean and standard deviation of 5 simulations with similar initial conditions. LB334, Lactobacillus paracasei; So34, Streptococcus oralis. (B) Final structure of a 16-h dual biofilm simulation. Red spheres represent *L. paracasei*, and green spheres represent S. oralis; extracellular matrix filled the space between cells but is not represented in the image for clarity. The surface area of this image is 18,496 μm^2^. (C to F) *In vitro* biofilm growth. (C) Biovolume plot for 16-h single- and dual-species biofilm cultures. LB334, Lactobacillus paracasei; So34, Streptococcus oralis. Plotted is the average biovolume from 18 microscopic fields, imaged from 4 wells in 2 independent experiments (So34 and dual) and from 22 fields from 5 wells in 3 experiments (LB334). (D to F) Three-dimensional reconstructions of a 16-h S. oralis mTeal biofilm (D), a 16-h *L. paracasei* (dyed with CellTracker red) biofilm (E), and a 16-h dual S. oralis*-L. paracasei* biofilm. S. oralis, left; *L. paracasei*, center; merged 2-channel image, right (F). Reconstructions were made in Imaris with the surfaces protocol from 63×, 14-bit images; image dimensions: 139.51 μm (968 pixels) by 104.92 μm (728 pixels). Z-slices = 20 (D), 19 (E), and 17 (F). Scale bar = 15 μm. ***, *P* < 0.001; ****, *P* < 0.0001.

We began by constructing a model where the only interactions between the two species were competition for space and for consumption of nutrients required for growth. Using the iDynoMiCS software (34), we constructed an agent-based model with the two bacterial species competing for glucose and oxygen. The growth parameters of the two species with respect to glucose were initially obtained from the literature (38, 39). However, the biofilm model using these parameter values behaved differently than the experiments; specifically, the simulated biovolume was only weakly affected by medium dilutions, while in the experiments this effect was stronger (see Fig. S1 in the supplemental material). Thus, we adjusted the maximum growth rate (μ_max_) and Substrate Utilization Constant for glucose ($K_{S_{g}}$) parameters such that the simulation would display as similar a dependence on medium concentration as the experiments. The growth parameters of each species with respect to oxygen were taken from the literature (34, 40). Simulations seeded with an initial density of 0.01 cells/μm^2^ of each species, as used in experiments, led to biofilms with the properties depicted in Fig. 1A and B.

10.1128/msphere.00656-22.1 FIG S1 *In vitro* biofilm growth of S. oralis (green) and *L. paracasei* (red) in biofilm media and medium dilutions from full strength to 0.10× (10% medium, 90% PBS). Biovolume was measured using Imaris software, from images taken after 16 h of growth at 37°C and 5% CO_2_. Lines represent linear regression with 95% confidence limits (dashed lines). Single-species cultures are solid symbols with a solid linear regression line; dual-species cultures are grey-filled symbols with dotted regression lines. Biovolumes were determined from 7 to 10 images from 4 biofilms per medium dilution in 2 experiments. Linear regression statistics: S. oralis in single-species biofilms: *y* = 7,762 × + 357.8, *R*2 = 0.7970; S. oralis in dual-species biofilms: *y* = 4,665 × – 795.1, *R*2 = 0.5978; *L. paracasei* in single-species biofilms: *y* = 4,401 × + 1,034, *R*2 = –.06542; *L. paracasei* in dual-species biofilms: *y* = –343.8 × + 2168, *R*2 = 0.008884. Download FIG S1, PDF file, 0.07 MB. Copyright © 2023 Archambault et al. 2023 Archambault et al. https://creativecommons.org/licenses/by/4.0/ This content is distributed under the terms of the Creative Commons Attribution 4.0 International license.

### Model predictions.

Agent-based models were set up to include each of the species in isolation, as well as a biofilm seeded with equal amounts of the two species. These models were then run to examine how dual biofilms would behave under the hypothesis of simple competition for space and nutrients. As can be seen in Fig. 1A, in single-species biofilms S. oralis is predicted to be a better biofilm former than *L. paracasei*, based on simulation biovolume estimates. As expected, there is a decrease in the biovolume of each species in the dual biofilm compared to the single biofilm, based on nutrient and oxygen competition. Interestingly, based on these parameters, the simulations predicted that in the dual biofilm the biovolume of S. oralis would be higher than that of *L. paracasei* (Fig. 1B).

A question arises whether the competition between the species is happening just for the space their cells occupy or if the carbon substrate is becoming limiting. To check this, we set up a control simulation where the two species have independent carbon sources (Substrate1 and Substrate2) and therefore are only competing for space. The results were very similar to the case in which they both use the same carbon substrate, so we conclude that in the conditions of the original simulation the two species only compete for space. Effectively, the fastest-growing species (S. oralis) controls more space than the slowest (*L. paracasei*). Details of the control simulation can be found in the supplemental material Text S1.

10.1128/msphere.00656-22.4 TEXT S1 Control model and sensitivity analysis. Contains information about the control model where the two bacteria compete for space and have two different carbon sources. It also contains the sensitivity analysis for the parameters calibrated for all the four models. Download Text S1, DOCX file, 0.02 MB. Copyright © 2023 Archambault et al. 2023 Archambault et al. https://creativecommons.org/licenses/by/4.0/ This content is distributed under the terms of the Creative Commons Attribution 4.0 International license.

### Live biofilm growth.

We next measured single and dual biofilm growth of S. oralis and *L. paracasei* experimentally to test the model predictions. Single- and dual-species biofilm cultures of S. oralis and *L. paracasei* acidify our biofilm media slightly; at the end of the 16-h growth period, the starting pH of 8 was reduced to pH 6.5. *L. paracasei* attained a lower biovolume than S. oralis when grown alone for 16 h, as predicted by the model. In coculture, *L. paracasei* growth was slightly, but not significantly, lower than that in single culture, while S. oralis biovolume was significantly reduced (Fig. 1C). This was different from the simulation, which predicted a similar reduction in growth for both species. Three-dimensional (3D) projections of biovolume made from images illustrate the growth pattern of S. oralis and *L. paracasei* in single- and dual-species biofilms (Fig. 1D to F). S. oralis biofilms take the form of interconnecting mounds of cells and reach similar heights when growing alone (averaging 8.9 ± 1.0 μm) (Fig. 1D) and when growing in dual-species biofilms (9.2 ± 0.9 μm) (Fig. 1F, left image; unpaired *t* test, *P* = 0.3890). *L. paracasei* biofilms were sparse and reached a thicknesses of 7.7 ± 2.0 μm and 7.9 ± 1.6 μm for single- and dual-species biofilms, respectively (Fig. 1E and F, middle image; unpaired *t* test, *P* = 0.6686). Because the 2 species formed biofilms with different configurations, we suspected there could be an imperfect correlation between biovolumes and cell counts. We therefore used genus- or strain-specific real-time quantitative PCR (qPCR) of 16S rRNA to estimate the number of each bacterial species that grew in the biofilms (Fig. S2B). The results of qPCR indicated that single-species biofilms of S. oralis contained more cells than single-species *L. paracasei* biofilms as predicted by the model. However, the reduction in S. oralis numbers was more pronounced than the reduction in *L. paracasei* numbers in dual-species than in single-species biofilms, and this contrasted with the model predictions. Overall, these experiments indicated that actual biofilm growth did not match the predictions of the first iteration of our model, in which competition for nutrients and space were the only interactions between the 2 species.

10.1128/msphere.00656-22.2 FIG S2 (A) Number of bacterial cells (agents) for single and dual biofilms from simulations of 16 h of growth of a purely competitive model. Results are expressed as the mean and standard deviation of 5 simulations with similar initial conditions. LB334, Lactobacillus paracasei; So34, Streptococcus oralis. (B) Cell numbers in *in vitro* biofilms grown for 16 h at 37°C and 5% CO_2_, as measured by qPCR targeting 16S rRNA gene for each species. S. oralis alone and S. oralis in dual biofilms were compared using the Mann-Whitney test; *, *P* = 0.0286. Other comparisons were not possible due to low sample numbers. Data are from 2 experiments; *n* = 4 biofilms of dual species and S. oralis alone and 2 biofilms of *L. paracasei* alone. These data are also presented in Fig. 3. Download FIG S2, PDF file, 0.03 MB. Copyright © 2023 Archambault et al. 2023 Archambault et al. https://creativecommons.org/licenses/by/4.0/ This content is distributed under the terms of the Creative Commons Attribution 4.0 International license.

### Exploration of the possible interactions within the biofilm.

Since we observed experimentally that there is a significant reduction in biovolume of S. oralis when it is grown with *L. paracasei* compared to when it is grown alone, we proposed two plausible explanations for these interactions:

1. *L. paracasei* product(s) may inhibit growth of S. oralis cells (41, 42).
2. *L. paracasei* may secrete a surfactant (27–29) that causes both cell types to detach from the biofilm.

In the model, the growth inhibition mechanism was incorporated by including the production of a bacteriostatic product by *L. paracasei* and its effect on slowing the growth of S. oralis. To include a surfactant mechanism in the simulations, we used a modified version of iDynoMiCS (36) in a way that allowed the biofilm cells of both bacteria to disperse from the biofilm surface based on the local surfactant concentration (see Materials and Methods). We also ran a model which incorporated both mechanisms. The simulations were repeated multiple times for the single and dual biofilm in all three models. A sufficient number of repetitions was run so as to achieve a coefficient of variation smaller than 10%. For the inhibition model, it was 5 runs, and for the models containing surfactant, it was 50.

**FIG 2:**
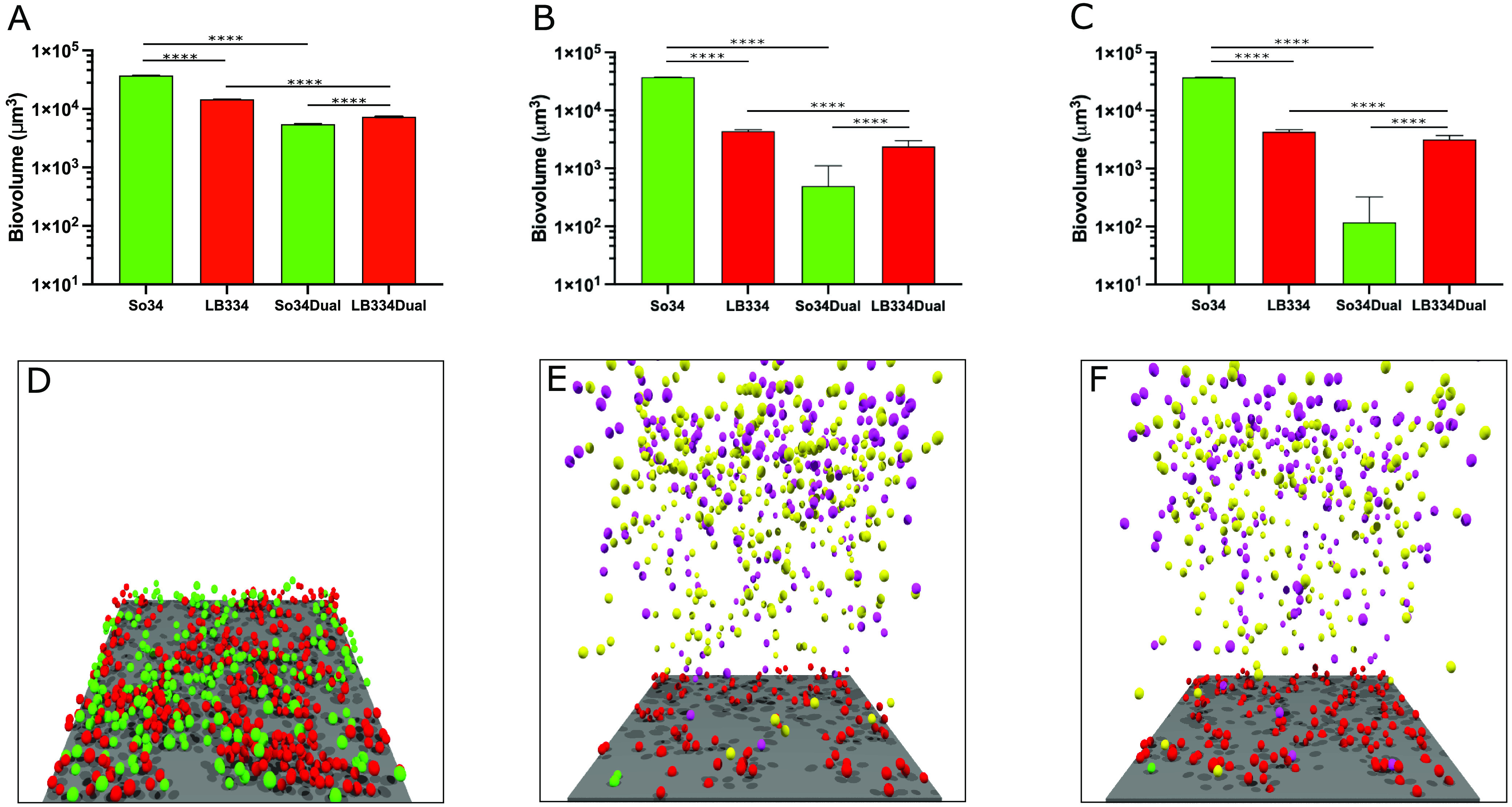
Simulation of biofilm growth in an inhibition, surfactant, and inhibition + surfactant model. (A) Biovolume plot for single and dual biofilms from the simulation of a 16-h biofilm with growth inhibition. LB334, Lactobacillus paracasei; So34, Streptococcus oralis. Results are expressed as the mean and standard deviation of 5 simulations with the same initial conditions as the competition model. (B) Biovolume plot for single and dual biofilms from the simulation of a 16-h biofilm with surfactants. The model was repeated 50 times to get a low coefficient of variation. (C) Biovolume plot for single and dual biofilms from the simulation of a 16-h biofilm with inhibition and surfactants. The model was repeated 50 times to get a low coefficient of variation. (D) Final structure of a 16-h dual inhibition model simulation. Red spheres, *L. paracasei*; green spheres, S. oralis; extracellular matrix filled the space between cells but is not represented in the image for consistency with surfactant images. The image with EPS in the inhibition model is available in the supplemental material (Fig. S3). The surface area of this image is 18,496 μm^2^. (E) Final structure of a 16-h dual-surfactant model. The magenta and yellow spheres floating above the biofilm are the planktonic cells of *L. paracasei* and S. oralis, respectively. (F) Final structure of a 16-h dual model with inhibition and surfactants.

In Fig. 2, the growth inhibition model (Fig. 2A), the surfactant model (Fig. 2B), and their combination (Fig. 2C) show that the biovolume of S. oralis is lower in the dual biofilm than the single biofilm. This suggests that either or both mechanisms could be responsible for the decrease in S. oralis biovolume in biofilms with *L. paracasei*. In the combination model, we see that the S. oralis cells nearly all detach from the biofilm, and thus its biovolume in the dual biofilm is close to zero. Figure 2D to F show the images of the simulated dual biofilm at 16 h after inoculation. The images in Fig. 2E and F, depicting the simulation of the models with surfactant production, also show the floating planktonic cells that had detached from the periphery of the biofilm. The planktonic cells of S. oralis and *L. paracasei* are a different color to distinguish the planktonic from the biofilm cells.

10.1128/msphere.00656-22.3 FIG S3 Simulation of biofilm growth in an inhibition, surfactant, and inhibition + surfactant model. (A) Biovolume plot for single and dual biofilms from the simulation of a 16-h biofilm with growth inhibition. LB334, Lactobacillus paracasei; So34, Streptococcus oralis. Results are expressed as the mean and standard deviation of 5 simulations with the same initial conditions as the competition model. (B) Biovolume plot for single and dual biofilms from the simulation of a 16-h biofilm with surfactants. The model was repeated 50 times to get a low coefficient of variation. (C) Biovolume plot for single and dual biofilms from the simulation of a 16-h biofilm with inhibition and surfactants. The model was repeated 50 times to get a low coefficient of variation. (D) Final structure of a 16-h dual inhibition model simulation. Red spheres, *L. paracasei*; green spheres, S. oralis; EPS is denoted by black (for S. oralis) and grey (for *L. paracasei)* spheres. The surface area of this image is 18,496 μm^2^. (E) Final structure of a 16-h dual-surfactant model. The magenta and yellow spheres floating above the biofilm are the planktonic cells of *L. paracasei* and S. oralis, respectively. (F) Final structure of a 16-h dual model with inhibition and surfactants. Download FIG S3, EPS file, 1.6 MB. Copyright © 2023 Archambault et al. 2023 Archambault et al. https://creativecommons.org/licenses/by/4.0/ This content is distributed under the terms of the Creative Commons Attribution 4.0 International license.

Because the simulations of the two hypotheses resulted in somewhat similar outcomes (at least qualitatively), we are not able to eliminate either of them as possible explanations of how S. oralis is affected by *L. paracasei* in mixed biofilms. Thus, we looked for experimental evidence for the action of either an inhibitory substance, a surfactant, or perhaps both. Our culture wells contained both attached cells (in the biofilm) and planktonic cells (removed with spent media before imaging), and we reasoned that a surfactant or an inhibitory substance might alter the number of planktonic and biofilm cells in different ways. We expected that a toxin released into the surrounding media could reduce the number of cells in both the biofilm and the planktonic phase, while a surfactant would tend to increase the proportion of planktonic bacteria without necessarily affecting the overall number of cells in each culture well. To determine the number of cells in each phase, we enumerated each species using qPCR with genus (for *Lactobacillus*)- or strain (for Streptococcus)-specific primers.

**FIG 3:**
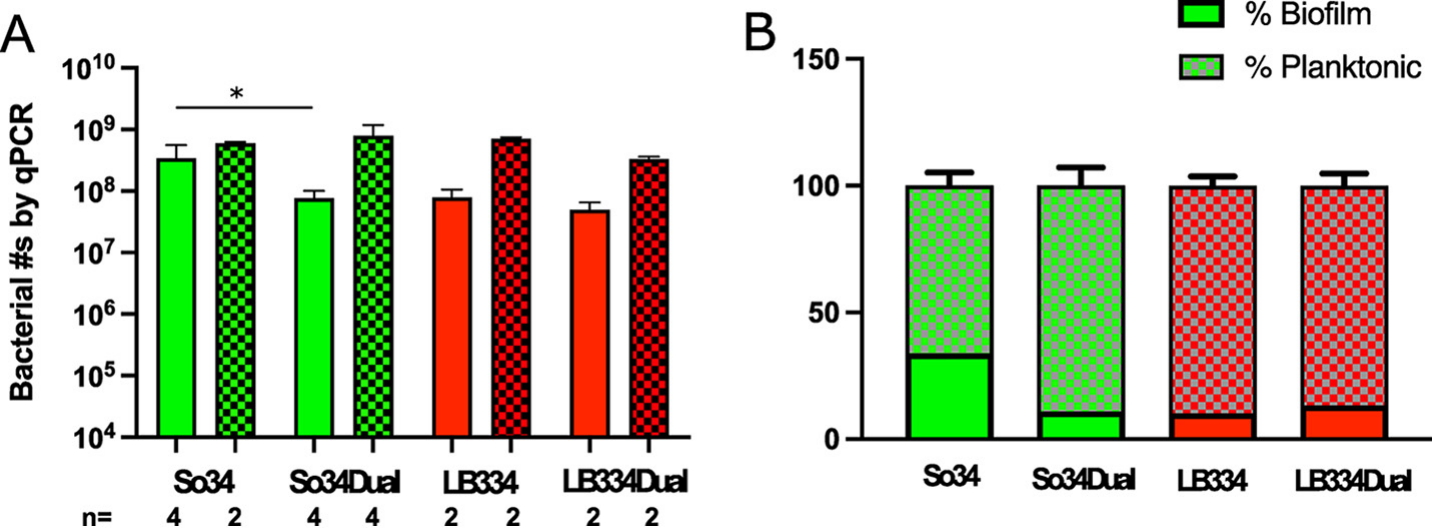
Experimental data demonstrate inhibition of S. oralis biofilm formation by *L. paracasei*. (A) Plot of bacterial numbers in biofilm (solid bars) and planktonic state (hashed bars) for single-species and dual-species biofilms determined by qPCR. Average of bacterial numbers from *n* biofilms; *n* as indicated in the figure. LB334, *L. paracasei*; So34, S. oralis. (B) Data from panel A plotted as a percentage of the total bacteria per well. S. oralis alone and S. oralis in dual biofilms were compared using the Mann-Whitney test; *, *P* = 0.0286. Other comparisons were not possible due to low sample numbers. Data are from 2 experiments; *n* = 4 biofilms of dual-species and S. oralis alone and 2 biofilms of *L. paracasei* alone.

The total number of S. oralis cells in the biofilm, as counted by qPCR, was reduced in the dual-species biofilms (Fig. S2B). However, the number of planktonic S. oralis cells was not reduced when *L. paracasei* was present (Fig. 3A). *Lactobacillus* numbers were similar for biofilms and for planktonic growth in single and dual-species cultures by this quantification method (Fig. 3A). These results suggest that growth inhibition of S. oralis by *L. paracasei* may occur principally in the biofilm growth phase.

We reasoned that a surfactant might increase the percentage of cells that detached from the biofilm, regardless of the overall growth of the bacteria in the well. To determine whether this occurred, the same qPCR data were plotted as a percentage of the total number of cells in each well (Fig. 3B). Interestingly, the percentage of S. oralis planktonic cells was higher in the mixed cultures than in the single cultures, while *L. paracasei* appeared to be unaffected, showing similar percentages of planktonic cells in single and dual cultures. Considering these results, the data suggest that the presence of surfactant activity is additionally plausible, indicating that more than one mechanism may be involved in the suppression of S. oralis biofilm growth in the presence of *L. paracasei*.

### Further experimental exploration of the system.

**FIG 4:**
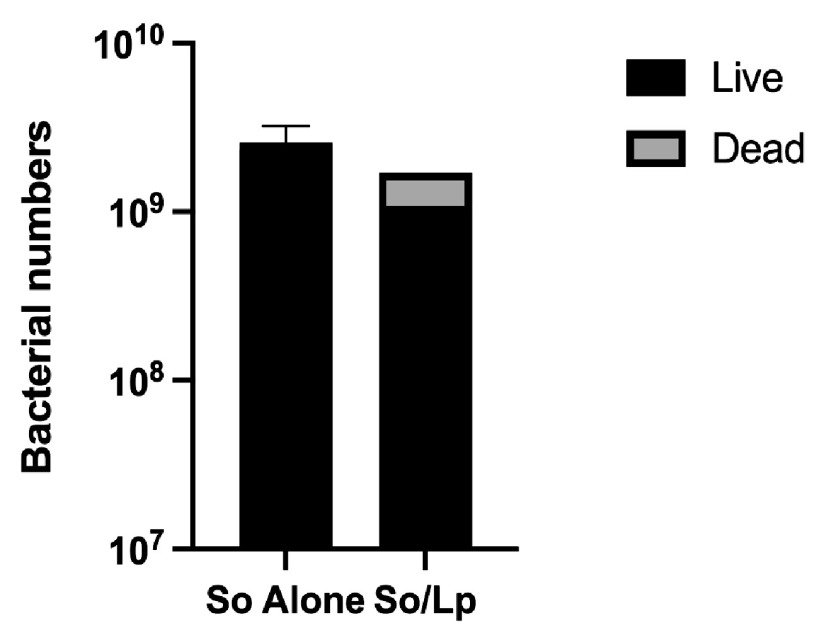
*L. paracasei* causes death of S. oralis in dual-species biofilms. S. oralis biofilms were grown alone or with *L. paracasei* for 16 h at 37°C and 5% CO_2_. Biofilm samples were split, and 1 set was treated with PMA. PMA enters nonviable cells and interacts with their DNA, preventing amplification in PCR. Live and dead S. oralis numbers in biofilms were determined by comparing qPCR results of PMA-treated and untreated samples. Results represent data from 2 biofilms (S. oralis alone) or 1 biofilm (dual-species).

Lactic acid bacteria, including *L. paracasei*, are known to produce peptides and proteins with antimicrobial properties collectively known as bacteriocins (43–45). To explore the possibility of cytotoxic activity by *L. paracasei*, we employed a viability qPCR method to determine the numbers of live and dead S. oralis cells in single- and dual-species biofilm cultures (Fig. 4) (46, 47). When growing alone, S. oralis experienced a low die-off rate of 9.17%, but in dual-species cultures, 36.51% of the total S. oralis DNA detected by qPCR was derived from dead cells. This result supports the hypothesis that *L. paracasei* produces a substance that acts as a bacteriocin against S. oralis in mixed-species biofilms.

## DISCUSSION

We have used a combined modeling and experimental process to construct and refine a model of dual-species biofilm growth which has, in turn, informed further experimental exploration of interspecies interactions in biofilms. Our initial agent-based model was limited to nutrient and space competition and did not fully explain the interaction between S. oralis and *L. paracasei.* To account for the differences between model and experimental results, we proposed two hypotheses for the inhibition of S. oralis biofilm growth: an inhibitory substance or a surfactant produced by *L. paracasei*. These hypotheses were translated into three different models, and simulations were carried out. In all three cases, the biovolume and cell numbers of S. oralis were reduced in the dual biofilm simulations compared to the single biofilm simulations, indicating that the experimental results could have been caused by either mechanism or both. Differences in S. oralis planktonic and biofilm growth in single- and dual-species biofilms *in vitro* also supported both mechanisms. We further explored the interaction and found that the presence of *L. paracasei* markedly increased the numbers of dead S. oralis cells in biofilm cultures. Prior to this study, *L. paracasei* and S. oralis interactions in biofilms had not been studied experimentally, and this is one of a few studies using a combination of agent-based models and experiments to study interspecies biofilm interactions.

Members of the viridans Streptococcus spp. have putative pathogenic roles, contrasted with the probiotic properties of *Lactobacillus* spp., and interactions between members of these genera have long been of interest to the medical community. For example, many *Lactobacillus* strains and their cell-free supernatants have antimicrobial activity against streptococcal pathogens, including S. mutans (23–25, 48) and S. pyogenes (26, 49). With a few notable exceptions (25), the mechanisms of inhibition are not fully understood. Interactions between Streptococcus and *Lactobacillus* are often species specific, and while most examples in the literature involve *Lactobacillus* spp. inhibition of Streptococcus spp., at least one example of the reverse has been found (50).

Surfactants are amphiphilic molecules that reduce surface tension and interfacial tension, thus interfering with adhesive interactions between microbes and the surfaces to which they attempt to attach. Biosurfactants produced by *Lactobacillus* spp. have been explored as a means of inhibiting biofouling in commercial applications and in prevention of biofilms growing on hard surfaces in the oral environment (22, 28, 29). Many studies have found biosurfactant activity in supernatants of *Lactobacillus* culture (18, 51–53). Many of the mixtures and molecules isolated from supernatants of *Lactobacillus* spp. have both antimicrobial and biosurfactant activities (22, 27–29, 54).

Streptococcus and *Lactobacillus* species are known to be both acidogenic and acid tolerant. As we reported, acidification occurs in our single- and dual-species biofilm cultures. However, the buffering capacity of the medium keeps the pH well above the lower limit for exponential growth of S. oralis: pH 4.14 to 4.88 for 12 Streptococcus species and strains, including S. oralis (55). Lower pH levels of 3.0 to 3.5 are required for killing of S. oralis (55). Therefore, it is unlikely that the pH of the culture media is responsible for the reported growth inhibition and death of S. oralis in dual-species cultures and when treated with *L. paracasei* spent-medium preparations. Overall, our experimental results support the presence of a substance with growth inhibition or bacteriocin-like properties produced by *L. paracasei* biofilm cultures, while the comparison between planktonic and biofilm-associated cells of S. oralis in dual cultures also hints at a mechanism that affected adhesion or dispersal of S. oralis.

Our agent-based model based in iDynoMiCS (34) is one of very few models which have been constructed with constant interaction and feedback with experimental work (36, 37). The growth parameters in the model were based on the growth of single-species biofilms in different concentrations of the media and prior published values. We investigated further interactions, namely, noncompetitive inhibition and surfactant effects, based on the observed interactions of S. oralis and *L. paracasei in vitro*.

The surfactant model was constructed using a modified version of iDynoMiCS (36) which incorporated the transition of biofilm cells to planktonic cells based on the local surfactant concentration. A limitation of the model was that the area considered was much smaller than that used in experiments, due to limitations on computational resources.

However, the ratio of the initial number of seed cells to surface area was the same in the experiments and simulations. Effectively, the simulations represent a small section of the biofilms grown experimentally but have similar characteristics in terms of biofilm thickness.

Interkingdom interactions such as those between oral bacteria and the fungus Candida albicans hold great interest due to medical treatments or immunocompromised states resulting in fungal-bacterial dysbiosis, a contributing factor in human disease (56). In certain host backgrounds S. oralis synergizes with C. albicans to increase the virulence of the fungus (5, 57–59). *L. paracasei*, on the other hand, inhibits the transition of C. albicans yeast to the hyphal form (23). Our next goal is to increase the complexity of our experimental and mathematical models by incorporating C. albicans to study interactions in three-species biofilms. An assumption made in this agent-based model is that all the cells (agents) are spherical in shape. One avenue of future research will be to incorporate the rod shape (for *Lactobacilli*) and filamentous shape (for fungi) into the software and observe the effect, if any, that these cell morphologies may have on the biofilm structure and dynamics. The insights gained through the iterative process of modeling and experimentation in our study of S. oralis and *L. paracasei* interactions during biofilm growth will guide us in exploring more complex, multispecies biofilms.

## MATERIALS AND METHODS

### Bacterial strains and culture methods.

Fluorescent S. oralis 34 teal (So34) (60, 61) from glycerol stocks was grown overnight in brain heart infusion (BHI) medium (Benton-Dickinson and Company, Sparks, MD, USA) supplemented with erythromycin, 5 μg/mL, as needed, under aerobic static conditions at 37°C with 5% CO_2_. *L. paracasei* ATCC 334 (LB334) was similarly cultured in De Man, Rogosa, and Sharpe (MRS) broth. Subcultures were grown from overnight cultures to an optical density reading at 600 nm (OD_600_ reading) of 1.0 (1 × 10^8^ cells/mL). Prior to biofilm growth, LB334 bacteria were labeled with CellTracker red CMTPX dye (Invitrogen, Carlsbad, CA, USA) according to the manufacturer’s protocol. Briefly, cells were diluted 1:10 in phosphate-buffered saline (PBS), centrifuged for 5 min at 2,000 × *g*, resuspended at a density of 1 × 10^7^ to 1 × 10^8^ cells/mL in 18 μM dye in PBS, and then incubated for 45 min at 37°C. The labeled cells were centrifuged, washed once in PBS, and resuspended in biofilm growth media. So34 cultures were diluted 1:10 in PBS. To satisfy the nutritional requirements of both bacterial species, biofilms were grown in an optimized complex medium containing 80% RPMI 1640 without l-glutamine, without phenol red (Thermo Fisher Scientific, Waltham, MA, USA), 10% BHI (BBL brain heart infusion, Benton, Dickson and Company, Sparks, MD, USA), and 10% FBS (fetal bovine serum, R&D Systems, Minneapolis, MN, USA) (56). Bacteria (1 × 10^6^) were seeded into μ-Slide 8-well chambered coverslips (IBIDI GmbH, Gräfelfing, Germany) and incubated at 37°C with 5% CO_2_ for 1 h; the supernatant containing unattached bacteria was removed and replaced with fresh media, and incubation continued for 16 or 24 h. Prior to imaging, media were removed and replaced with PBS.

### Microscopy and image analysis.

Biofilms were imaged on a Zeiss Axio Observer inverted microscope with Apotome2 (Carl Zeiss, Inc., Thornwood, NY, USA). Unless otherwise noted, images were made using a 63× oil immersion lens. Images were analyzed using Imaris software (Oxford Instruments plc, Abingdon, Oxfordshire, UK). The biovolume of biofilms was measured from 3D reconstructions using the “surfaces” protocol in Imaris. Briefly, to measure biovolumes, we manually set a threshold intensity which excluded background fluorescence and then created surfaces which represent the volume occupied by each bacterial species. Objects less than ~1 μm^3^ were excluded, and the output volumes were added to obtain the total biovolume per image.

### qPCR enumeration of biofilms.

Biofilms were grown as described above. Supernatants were removed at 1 h and 16 h, placed in sterile 2-mL tubes, and centrifuged for 5 min at 10,000 × *g*, and pellets were frozen at −80°C. Biofilm starting cultures (1 h) and mature (16 h) biofilms were frozen in their imaging well slides immediately after imaging. DNA extraction was carried out using the DNeasy blood and tissue kit (Qiagen, Germantown, MD, USA) according to the manufacturer’s instructions, including the suggested pretreatment with enzymatic lysis buffer for Gram-positive bacteria. Genomic DNA was eluted in 100 μL nuclease-free water. qPCR was carried out using S. oralis 34 strain-specific primers (*wefA* to *wefH*; forward, 5′-CATCAAGAACTTCTCGGAGTTG-3′; reverse, 5′-CCACAGCTCCAGAATATTTAGC-3′) (57) and all lacto primers (forward, 5′-TGGATGCCTTGGCACTAGGA-3′; reverse, 5′-AAATCTCCGGATCAAAGCTTACTTAT-3′) and TAMRA probe, ACAAGCTATGAATTCACTTGC (62). For viability PCR, single- and dual-species biofilms were grown as described above except in 1 mL of medium in 24-well culture plates (Corning). Propidium monoazide (PMA)-qPCR was performed according to the manufacturer’s instructions (Biotium, Fremont, CA, USA). Briefly, biofilms were removed and homogenized by scraping, pipetting, and vortexing. Samples were split, and one portion was treated with PMA and the other portion was treated with deionized water and incubated 10 min in the dark. Samples were exposed to LED light for 15 min in a PMA-Lite box (Biotium). DNA extraction and qPCR were then carried out as described above.

### Data analysis and statistics.

The biovolume and qPCR data were graphed, and statistical analyses were carried out in Prism9 (GraphPad Software, San Diego, CA, USA). Live biofilm data were analyzed by ordinary one-way analysis of variance (ANOVA) with Sidak’s multiple-comparison test.

### Agent-based model and simulations.

All simulations shown were carried out with the iDynoMiCS software (34), which can run agent-based simulations of biofilms including multiple species. The models in this paper include two bacterial species, S. oralis and *L. paracasei*, which are represented by two different types of agents in iDynoMiCS. These agents are governed by rules that represent cell growth, cell division, cell death, production of extracellular polymeric substances (EPS), cell shoving, and cell detachment from the biofilm (34). The full ODD (overview, design, details) protocol according to Grimm et al. (63) for all the agent-based models is included in Text S2. The software also simulates medium nutrient diffusion and a liquid phase above the biofilm. Postprocessing of simulation results was carried out with R (64) scripts, and images were rendered with PovRay (65) software. All the relevant code and the iDynoMiCS protocol files for the different models can be obtained from https://github.com/skoshyc/StrepLactoBiofilmModeling.

10.1128/msphere.00656-22.5 TEXT S2 ODD (overview, design, details) protocol according to Grimm et al. (63) for all the agent-based models. Download Text S2, DOCX file, 0.07 MB. Copyright © 2023 Archambault et al. 2023 Archambault et al. https://creativecommons.org/licenses/by/4.0/ This content is distributed under the terms of the Creative Commons Attribution 4.0 International license.

The models presented here are three dimensional, with a computational grid of 136 by 136 by 136 μm in size. Only parameters related to growth, surfactant and toxin production, the inhibitory constant, and tolerance to surfactant were calibrated. A full sensitivity analysis was carried out for these parameters and is included in Text S1. All the other parameters were kept at default iDynoMiCS values (34).

The Monod growth rate for each cell size is given by:

$$\frac{\mathrm{dX}}{\mathrm{dt}}=\mu_{\max}\frac{S_{g}}{K_{S_{g}}+\;S_{g}}\frac{S_{o}}{K_{S_{o}}+\;S_{o}}X$$

where X is the biomass of the cell. The two nutrients considered here are glucose and oxygen, and their concentrations are denoted by *S_g_* and *S_o_*, respectively. μ_max_ is the maximum specific growth rate with unit 1/h, and *K_S_* (grams/liter) is the value of the substrate *S* when the specific growth rate is μ_max_/2.

### Estimation of growth parameters.

The growth parameters specific to glucose (namely, μ_max_, $K_{S_{g}}$) obtained from the literature were for the biofilm growth of Streptococcus gordonii (39) and the growth of Lactobacillus casei in a bioreactor (38). Using these literature parameter values, we then ran 16-h simulations of single-species biofilms for different glucose concentrations (2 g/L, 1.5 g/L, 1 g/L, 0.5 g/L, 0.2 g/L, 0.1 g/L, 0.02 g/L). The initial value of 2 g/L was set to match the sugar present in the biofilm media used in our experiments. On running simulations for different glucose concentrations, it was observed that the biovolume of the species S. oralis and *L. paracasei* did not reduce with a reduction in glucose, in contrast to what was observed in biofilm experiments (Fig. S1). We assumed a biologically relevant range of the glucose growth parameters (namely, μ_max_ and $K_{S_{g}}$). We then optimized the parameters within that range (one at a time using the bisection method; see, e.g., reference 66) to obtain similar reductions in biovolume upon reducing the carbon source similarly to our experiments. The parameter values which gave rise to biovolume reduction with respect to glucose dilution are the ones listed in Table 1. The parameter values with respect to oxygen (i.e., $K_{S_{o}}$) for each species were as in the literature (34, 40). The final parameter values used in all the simulations are displayed in Table 1.

**TABLE 1:** Parameter values used for cell growth of the two species in the biofilm simulations

Parameter	S. oralis	*L. paracasei*
Initial glucose concn, *S_g_* (g/L)	2	2
Initial oxygen concn, *S_o_* (g/L)	0.0064	0.0064
Μ_max_ (1/h) for growth	0.32	0.153
*K_Sg_* (g/L)	1.756	1.2
*K_So_* (g/L)	0.192e-3	0.2e-3
*K_I_* (g/L)	0.0025	
Yield_glucose	−3	−0.17
Yield_oxygen	−2	−1
Biomass:capsule	0.8:0.2	0.9:0.1
*K* (1/h) of production of toxin and surfactant by *L. paracasei*		0.7
Yield of inhibitor by *L. paracasei* (g/g)		0.3
Yield of surfactant by *L. paracasei* (g/g)		0.4

All biofilm simulations were started with a seed of 176 cells. In the case of mixed-species biofilm simulations, there were 88 cells of each type, such that the same total number of cells is kept constant. This matches the experiments which seeded the biofilms at 0.01 cells/μm^2^.

### Noncompetitive inhibition.

In this case, the simulation includes secretion by *L. paracasei* of an inhibitor of the growth of S. oralis through noncompetitive kinetics. This required including the term $\frac{K_{I}}{K_{I}\;+\;I}$ in the growth rate equation of S. oralis*:*

$$\frac{\mathrm{dX}}{\mathrm{dt}}=\mu_{\max}\frac{S_{g}}{K_{S_{g}}\;+\;S_{g}}\frac{S_{o}}{K_{S_{o}}+\;S_{o}}\frac{K_{I}}{K_{I}\;+\;I}X$$

where *K_I_* is the concentration of the inhibitory substance *I* that gives rise to a specific growth rate of μ_max_/2.

**FIG 5:**
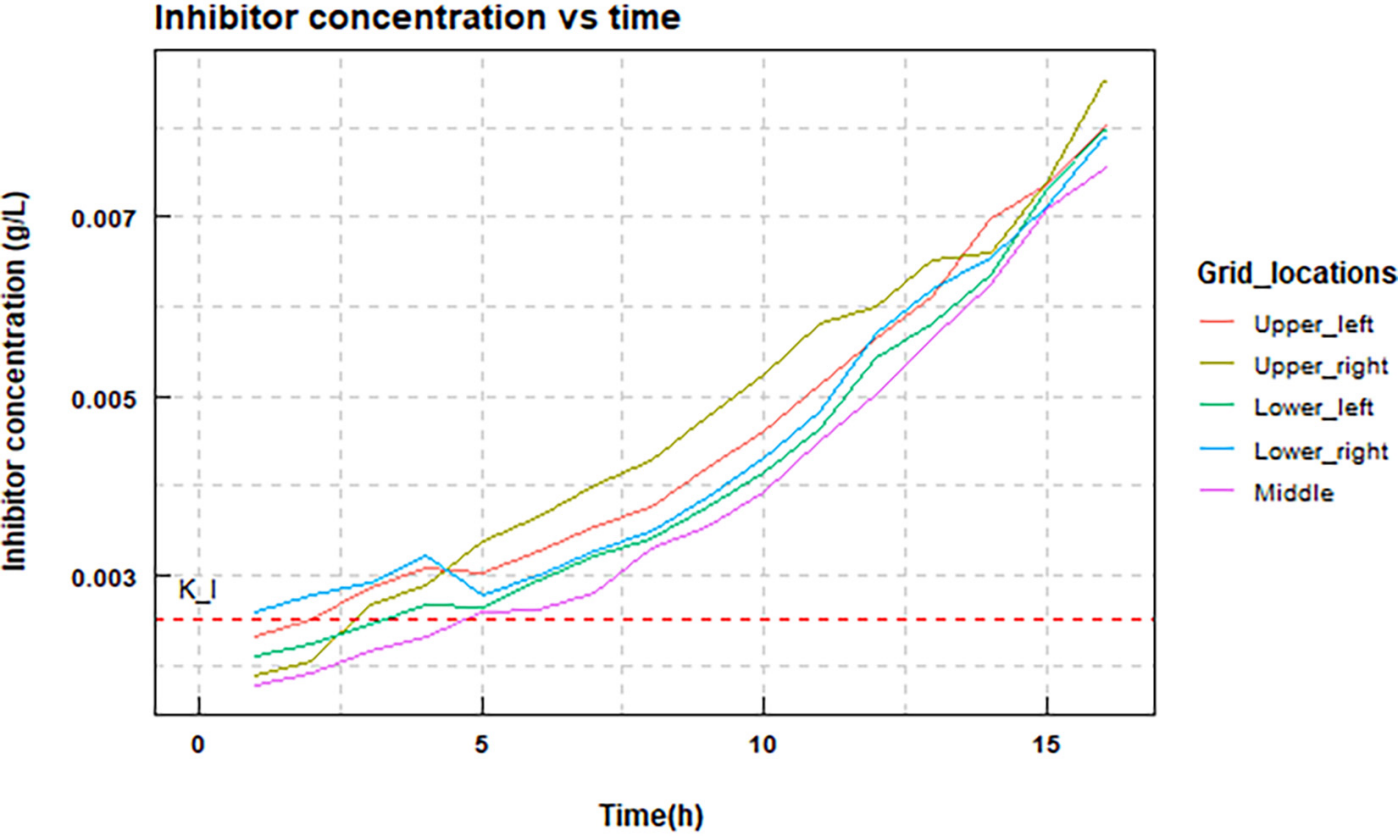
Inhibitor concentration in a dual biofilm at different time points in the simulation. Data were sampled at different locations of the biofilm, the four corners and the center of the surface area covered by the simulation. The red dashed line is the value of *K_I_* = 0.0025, which shows that the inhibitory effect of the toxin on S. oralis is present from around 5 h.

Since this is a hypothetical inhibitor, we cannot match its parameters to any real data. What is important is to have the *K_I_* for S. oralis be around the concentration of that substance in the biofilm, so as to effectively cause an inhibition (Fig. 5). The inhibitor production is given as a separate first-order reaction with *k* = 0.7 (1/h) with a yield of 0.3 of the inhibitor. We set *K_I_* arbitrarily to 0.0025.

### Surfactant simulation.

To simulate the action of a surfactant, we utilized the software from reference 36 that is a modified version of iDynoMiCS 1.1 software. The original intent of their modification was to include secretion of a chemotaxis agent (36). In that modification the chemotaxis agent repels Helicobacter pylori cells which detach from the biofilm. For our purposes, the surfactant produced by *L. paracasei* plays a similar role to that repellant; however, the effect is now on both cell types because the action of the surfactant is of a physicochemical nature affecting both cell types (though not necessarily to the same degree, since that could be determined by their cell wall composition). The surfactant is produced by *L. paracasei* cells, and when its concentration becomes higher than a threshold, it causes cells of both species to detach from the biofilm and become planktonic. Any cell in the periphery of the biofilm is subject to detachment based on the concentration of surfactant in its vicinity (36). The planktonic cells are in the boundary layer right below the bulk medium (36). In the model, exopolysaccharide (EPS) production is not included.

**FIG 6:**
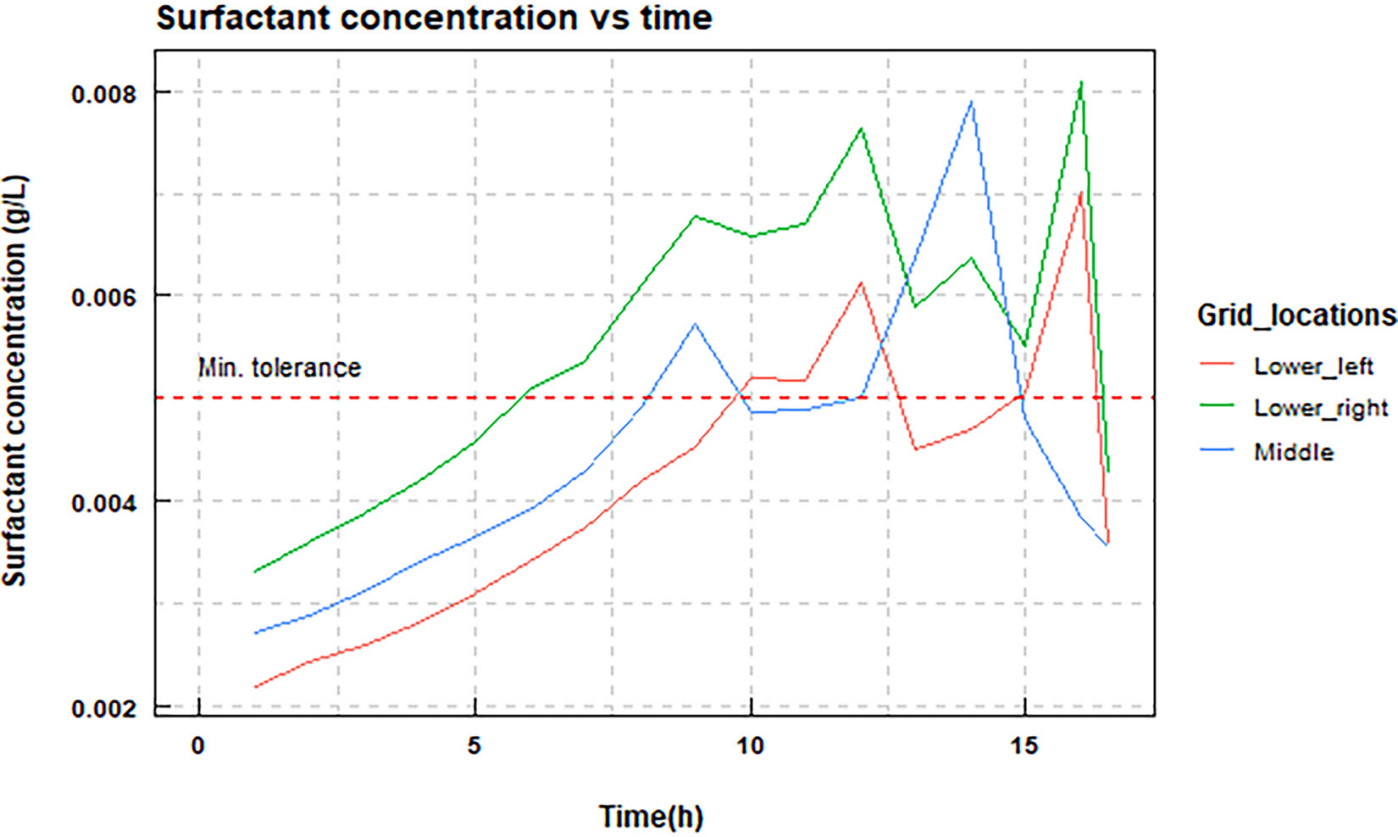
Surfactant concentration in a dual biofilm at different time points in the simulation. Data were sampled at different locations of the biofilm, the lower corners and the center of the surface area covered by the simulation. The red dashed line is the value of 0.005, which is the minimum of the tolerance thresholds for each of the species.

Like in the inhibition model, we do not have real data on the tolerance levels of the bacteria to the surfactants. We assume that the *L. paracasei* require higher concentrations of surfactant before detaching from the biofilm, as the biosurfactant is meant to be more antiadhesive to other pathogens (27). The tolerance threshold of surfactant for *L. paracasei* is 0.008 g/L and for S. oralis is 0.005 g/L. The thresholds were chosen based on the production of the surfactant by *L. paracasei* and were high enough to show a moderate effect (Fig. 6). The surfactant production is given as a separate first-order reaction with *k* = 0.7 (1/h) with a yield of 0.4.

Unlike in the chemotaxis simulation of Sweeney et al. (36), we assume that the planktonic cells do not rejoin the biofilm. To visualize this detachment in the simulation results, detached cells are depicted in a different color than the biofilm cells (Fig. 2E and F). The software includes random movement of planktonic cells in the liquid phase. The modified software that we used is available from https://github.com/alexwweston/iDynoMiCS.

We ran simulations of 16 h of growth for both single- and dual-species biofilms to see the effect of surfactants on the biofilm characteristics. We also ran simulations of the effect of the inhibitory substance and the surfactant together on the dual-species biofilms. In all cases, statistics are provided of multiple simulations with the same input parameters, due to the stochastic nature of the simulations. The number of simulations for the competition and inhibition model was five, and for the models with the surfactant the number was 50. We chose the number of simulations to achieve a coefficient of variation value of 10%. The coefficient of variation was estimated for species biovolume and count.

For each of the simulation models, the biovolume and species count were estimated using modified functions from the R package iDynoR (67). The average biovolumes of the single and dual biofilms were compared using the Games-Howell test in the R package ggstatsplot (68).

### Data availability.

All the relevant code and the iDynoMiCS protocol files for the different models can be obtained from https://github.com/skoshyc/StrepLactoBiofilmModeling.
