# Supplementary figures and images for: Corrected and Republished from: “Understanding Lactobacillus paracasei and Streptococcus oralis Biofilm Interactions through Agent-Based Modeling”
Source: mSphere. 2023 Mar 21;8(2):e00656-22. doi: 10.1128/msphere.00656-22 (PMC10187049; doi:10.1128/msphere.00656-22)

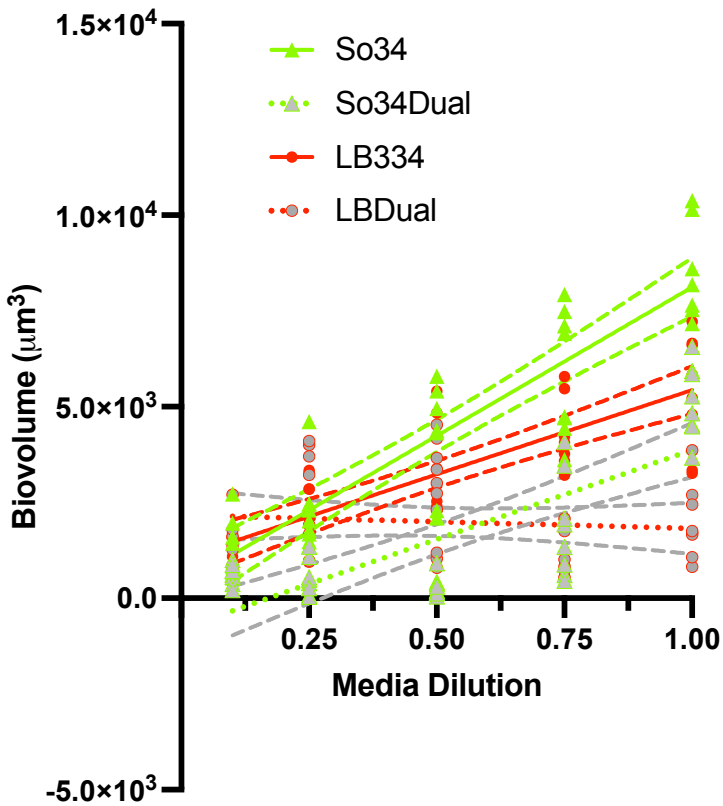

Supplement: FIG S1 [file msphere.00656-22-s0001.pdf]

**A**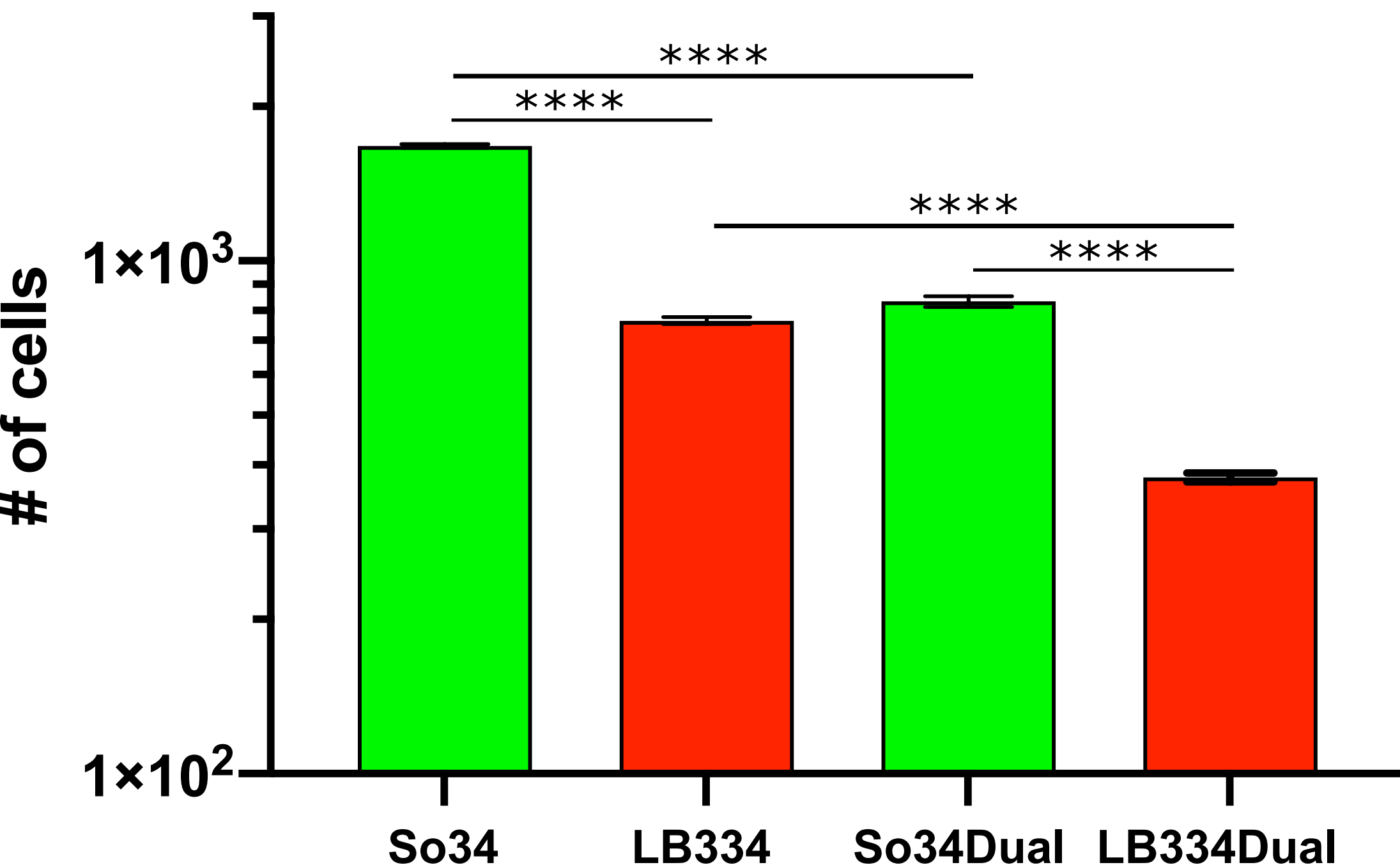**B**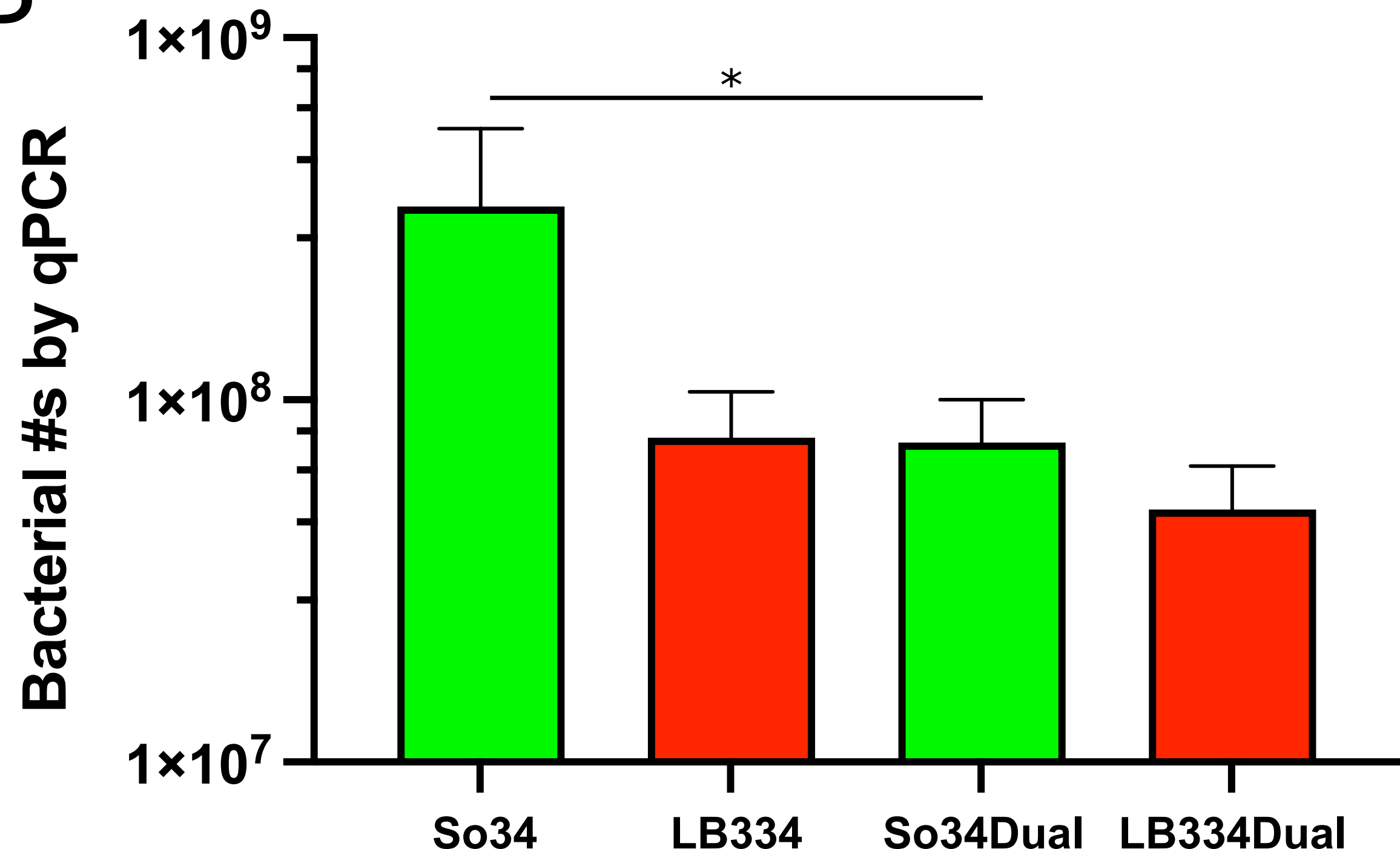

Supplement: FIG S2 [file msphere.00656-22-s0002.pdf]
